# Cleaned Meta Pseudo Labels-Based Pet Behavior Recognition Using Time-Series Sensor Data

**DOI:** 10.3390/s24113391

**Published:** 2024-05-24

**Authors:** Junhyeok Go, Nammee Moon

**Affiliations:** Department of Computer Science and Engineering, Hoseo University, Asan-si 31499, Republic of Korea; junhyeok970306@gmail.com

**Keywords:** cleaned meta pseudo labels, behavioral recognition, time-series data analysis, CNN, sensor data

## Abstract

With the increasing number of households owning pets, the importance of sensor data for recognizing pet behavior has grown significantly. However, challenges arise due to the costs and reliability issues associated with data collection. This paper proposes a method for classifying pet behavior using cleaned meta pseudo labels to overcome these issues. The data for this study were collected using wearable devices equipped with accelerometers, gyroscopes, and magnetometers, and pet behaviors were classified into five categories. Utilizing this data, we analyzed the impact of the quantity of labeled data on accuracy and further enhanced the learning process by integrating an additional Distance Loss. This method effectively improves the learning process by removing noise from unlabeled data. Experimental results demonstrated that while the conventional supervised learning method achieved an accuracy of 82.9%, the existing meta pseudo labels method showed an accuracy of 86.2%, and the cleaned meta pseudo labels method proposed in this study surpassed these with an accuracy of 88.3%. These results hold significant implications for the development of pet monitoring systems, and the approach of this paper provides an effective solution for recognizing and classifying pet behavior in environments with insufficient labels.

## 1. Introduction

Recently, due to urbanization and changes in lifestyle, there has been a sharp increase in the number of households with pets [1,2]. These changes have led to closer relationships between pets and humans, and an increased interest in the health and welfare of pets in pet-owning households [2]. As the demand for pet health management and behavior monitoring increases, various pet care services are being developed [3,4,5]. In particular, advancements in wearable technology and the Internet of Things (IoT) have opened new possibilities for monitoring everyday activities and health conditions across various fields [6,7,8].

Despite these technological advancements, the process of effectively collecting and analyzing pet behavior data still faces numerous challenges. The process of collecting sensor data using wearable devices is influenced by various factors such as pets’ uncooperative behavior, discomfort from wearing devices, and changes in the external environment. Additionally, the complexity of irregular and unstable time-series data complicates data interpretation, and many studies are using artificial intelligence to address this complexity of time-series data [9,10,11]. However, the high cost in terms of time and manpower required for labeling large data sets serves as an additional major factor that constrains the progress of research in pet behavior recognition.

Therefore, this study utilizes a meta pseudo labels-based self-training method as a way to improve the accuracy of pet behavior recognition and reduce the cost of data labeling [12]. Self-training methods, which effectively train the entire data set using a small amount of labeled data, have been explored in various attempts [13,14,15,16,17]. However, these methods are mainly utilized in image classification and lack the processing for the time-series characteristics of sensor data, which leads to the inability to exclude the noise of inactive data during the learning process. This study aims to remove inactive data using the cleaned meta pseudo labels method, which additionally utilizes the loss representing the distance between the extracted features and the center values updated during learning.

This paper developed a behavior recognition model based on sensor data collected from wearable devices equipped with 3-axis accelerometers, gyroscopes, and magnetometers, including 3-axis rotation values. By labeling only a portion of the collected data to reduce costs and integrating the learning of labeled data and unlabeled data through the meta pseudo labels technique, inactive data from unlabeled data were eliminated using the features of the labeled data. The behaviors are classified into five categories: standing, walking, sitting, lying down, and eating.

The structure of this paper is as follows: Section 2 reviews existing studies on pet behavior recognition and self-training techniques. Section 3 describes in detail the structure and learning process of the pet behavior recognition model proposed in this study. Section 4 analyzes the experimental design and results of the proposed model in detail. Finally, Section 5 discusses the research findings and directions for future research improvements.

## 2. Related Work

### 2.1. Behavior Recognition

In services aimed at improving the living standards and health management of pets, behavior recognition has become an essential component. Moving away from the initial reliance on video data or the need for manual input by users, modern systems integrate video processing, deep learning, and sensor-based monitoring to automatically recognize and classify the behavior patterns of pets [18,19]. Particularly, research on sensor-based monitoring technologies, which do not raise concerns about privacy invasion or violation of personal space, is being actively conducted [3,19,20,21].

Recent studies on sensor-based monitoring are exploring new approaches that integrate various types of sensors into wearable devices to monitor pet behavior in real-time. These studies primarily utilize 3-axis accelerometers and gyroscope sensors to collect data, which is then used to train deep learning models [19,20,21]. Specifically, algorithms like convolutional neural networks (CNNs) and recurrent neural networks (RNNs) are employed to effectively learn the time-series characteristics of sensor data and accurately recognize complex behavior patterns.

However, behavior recognition still faces several challenges, including the high cost of data labeling, recognizing subtle differences between various behaviors, and the generalization issue regarding the behavior diversity across different species and individuals of pets. These issues reduce the performance and reliability of behavior recognition systems, making the development of monitoring challenging.

To address these challenges, this study developed a wearable device capable of additionally collecting magnetometer sensor data and rotation values, in addition to 3-axis accelerometer and gyroscope data. This wearable device transmits data to mobile devices in real-time via low-power Bluetooth, and the collected data undergo a preprocessing process to remove missing data and fuse information between multiple sensors to increase the accuracy of learning. Furthermore, to overcome the issue of high data-labeling costs, a self-training method based on meta pseudo labels was employed.

### 2.2. Self-Training in Time-Series Data

Deep learning models based on supervised learning require a large amount of data, which particularly presents a problem in terms of labeling costs and efficient use of resources in large data sets. Therefore, instead of additional data collection, new learning strategies aiming to maximize the use of existing data have been sought. In this context, semi-supervised learning is used as an alternative [22]. It focuses on expanding the training data set and improving the generalization ability of the model by simultaneously utilizing a small number of labeled data and a large volume of unlabeled data. However, semi-supervised learning methods, which often rely heavily on specific architectures or algorithms, can sometimes limit the flexibility in model design.

To overcome these limitations, self-supervised learning has been explored [14]. Initial self-supervised learning methods started with a simple approach where the model uses artificially generated pseudo labels for training [13]. However, this approach can lead to the potential distortion of learning due to errors in pseudo labels. One solution to this problem has been the study of methods using meta pseudo labels, which improve the quality of pseudo labels through continuous feedback and interaction between the teacher model and the student model [9,23,24]. This method has recorded high performance in image classification and has shown better performance than supervised learning depending on the data. In particular, meta pseudo labels have been used in combination with unsupervised data augmentation (UDA) to further enhance the generalization performance of the teacher model using a strategy [9,25,26].

In this study, cleaned meta pseudo labels based on meta pseudo labels were used. Recognizing the problem that inactive data included in unlabeled data could affect model performance, an embedding space was created to allow the teacher model to classify inactive data as a separate class when generating pseudo labels, thereby eliminating inactive data.

## 3. Cleaned Pseudo Labels-Based Pet Behavior Recognition

**Figure 1:**
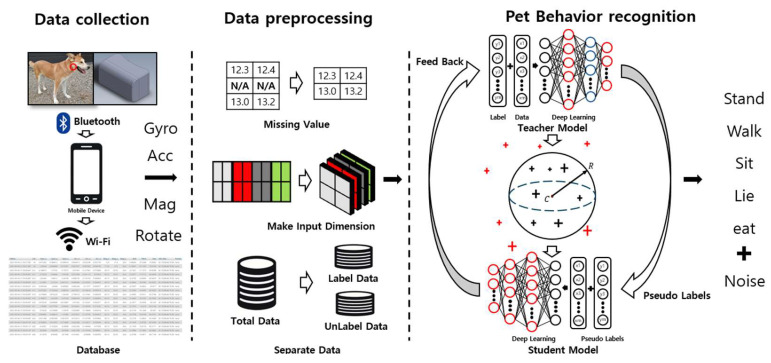
Process of cleaned pseudo labels-based pet behavior recognition.

The entire process of cleaned meta pseudo labels-based pet behavior recognition in this paper is as illustrated in Figure 1.

Data are collected through a wearable device that can be worn by pets. These data are transmitted to a mobile application via Bluetooth and Wi-Fi and are ultimately stored in a database. The stored data undergo preprocessing into a multi-dimensional structure to convert them into a format that the model can understand more effectively. During this process, preprocessing tasks such as handling missing values are performed to ensure optimal data quality. Subsequently, these data are divided into labeled and unlabeled data for training. In the next step, the divided data are utilized as training data for the teacher model and the student model, which apply the meta pseudo labels method. These models extract features from the data and use these to predict various behaviors of pets. The teacher model is used to generate pseudo labels, and the student model learns from these pseudo labels. During this process, the distance between feature values and the center values is measured, identifying and removing data considered as noise. Through this, the model classifies five types of behaviors: when the pet is standing, walking, sitting, lying down, or eating.

### 3.1. Data Collection

**Figure 2:**
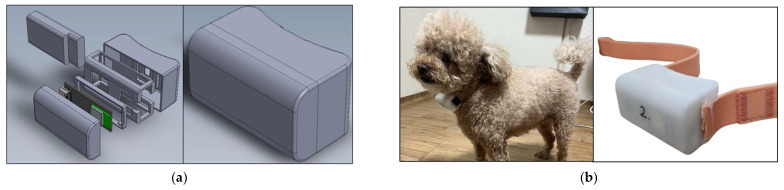
(**a**) CAD images of wearable sensors. (**b**) Physical devices and worn images.

In this study, sensor data including accelerometers, gyroscopes, magnetometers, and the 3-axis rotation angles of roll, pitch, and yaw were collected at a frequency of 50 Hz. The wearable device used for data collection incorporated an IMU (ICM-20948), an MCU (MAX32 670GTL), and a PMU (MAX77734), and the collected data were transmitted to a mobile device via Bluetooth. The structure of the wearable device is as shown in Figure 2. Concurrently, for accurate labeling, videos were also recorded using the camera of the mobile device. Both the sensor and video data collected in this manner were uploaded to a web server’s database. Data were collected from a total of 13 animals in various indoor and outdoor environments. The data collection was conducted in the presence of a companion for the pets’ safety. The wearable device was designed to be comfortably worn by dogs and placed under the neck, a position that is difficult for them to remove or damage. Panel (a) represents the CAD image of the wearable device custom-made for data collection, while (b) shows the actual image of the wearable device and an image of a dog wearing it.

### 3.2. Data Preprocessing

#### 3.2.1. Remove Missing Values

One of the inevitable issues during data collection using wearable devices is missing data. Especially when transmitting data via wireless communication technologies like Bluetooth, data loss can occur due to connection instability or interference, leading to consecutive missing values. These missing values can cause serious problems in data analysis and model training and can be particularly detrimental in self-supervised learning methods like meta pseudo labels. The meta pseudo labels method relies on a small set of labeled data, which means the accuracy of the data and labels directly and significantly impacts the model’s performance during the learning process. Therefore, simply deleting data containing missing values might be the best solution. Especially in research where the accuracy and reliability of labeled data are crucial, securing high data quality through data deletion may be more desirable than applying complex interpolation techniques. By excluding data containing missing values from the collected data set during the training process, the accuracy and reliability of the labeled data were ensured.

#### 3.2.2. Data Normalization

Normalization adjusts all features in a data set to share a common scale, preventing the model from overly depending on one part of the data. This normalization process is essential, especially when dealing with data that have different units or ranges. The collected data have ranges of −16.4 to 16.4 for Gyro, −2048 to 2048 for Acc, −2000 to 2000 for Mag, and −2000 to 2000 for Rotate. Therefore, Z-Score normalization was used for normalization. Z-Score is a method of scaling data according to the standard normal distribution, normalizing each feature in the data set so that the mean is 0 and the standard deviation is 1. This ensures all features are on the same scale, preventing the model from becoming overly dependent on specific features.

#### 3.2.3. Data 4D Reshaping

**Figure 3:**
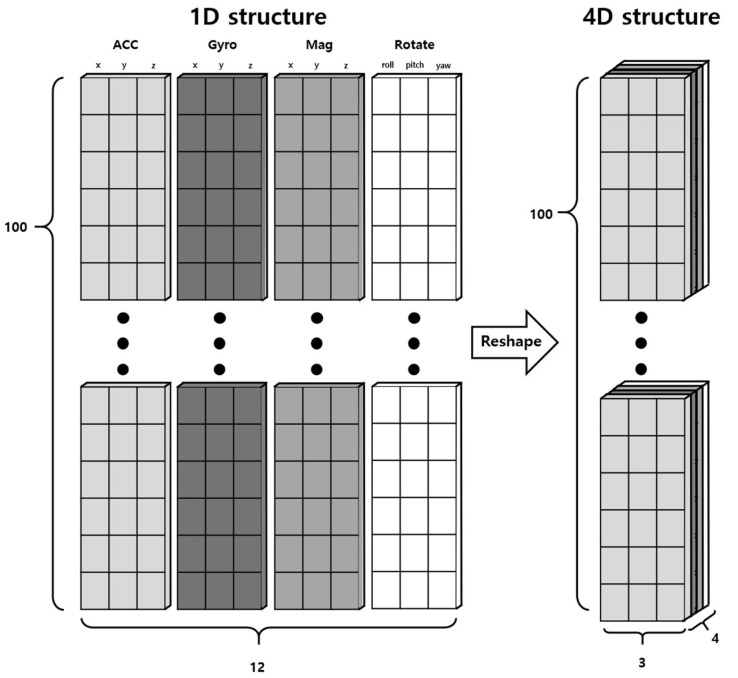
Process of preprocessing from 1-dimensional data to 4-dimensional data.

The sensor data collected in this study, comprising Gyro, Acc, Mag, and Rotate, utilized a wider range of sensor values compared to previous research. The movements of pets are complex and unpredictable, leading to the expectation of deep interrelations among the data collected from the sensors. These interrelations can be better captured through a 4-dimensional structure rather than a simple 1-dimensional structure, allowing for a more effective extraction of common features among the sensors. Consequently, the conventional sequence data structure of (100, 12) was transformed into a 4-dimensional data structure of (100, 4, 3), as illustrated in Figure 3, to be used as the input for the behavior recognition model. Such transformation allows for better structuring of each sensor’s data and learning of the interactions between sensor data.

### 3.3. Cleaned Meta Pseudo Labels-Based Pet Behavior Prediction

This study proposes a model for pet behavior recognition based on pseudo labels. This system, building on the existing meta pseudo labels method, introduces a novel approach by excluding inactive data during the learning process and re-labeling it as noise. This method is optimized for sequential data characteristics, allowing for effective recognition of diverse pet behavior patterns.

Furthermore, this study enhances the model’s performance by incorporating the UDA strategy. UDA increases data diversity through Weak Augmentation and Strong Augmentation, thereby improving the accuracy of the pseudo labels generated by the teacher model.

**Figure 4:**
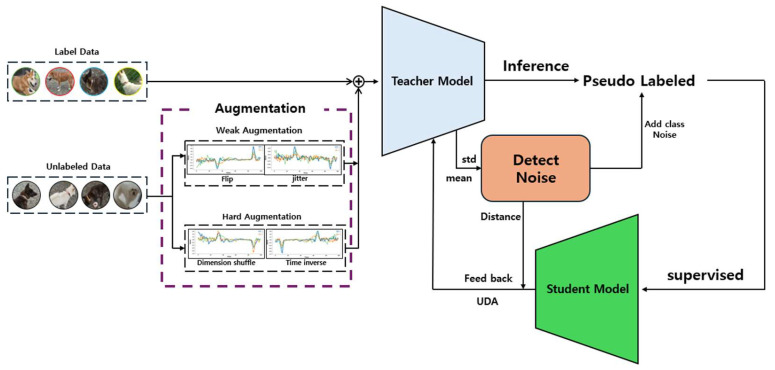
Cleaned meta pseudo labels-based learning process.

**Figure 5:**
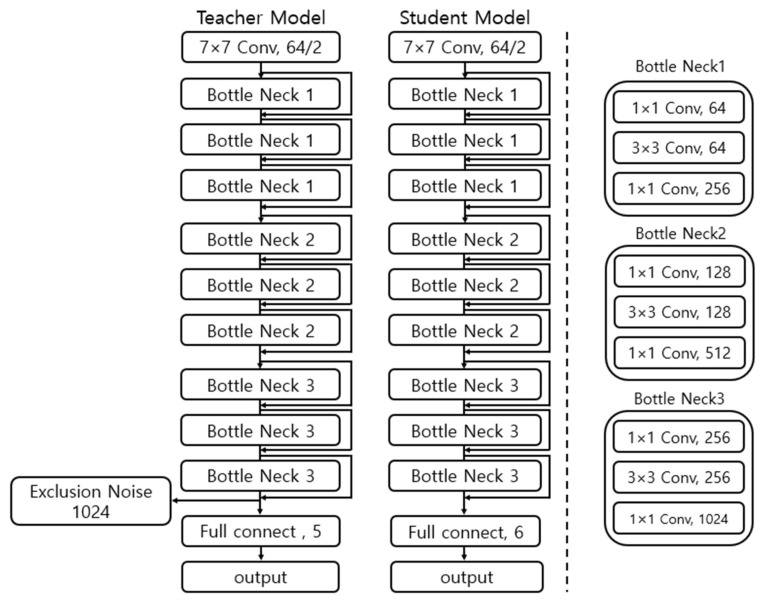
Teacher and student model structures based on pseudo labels.

The proposed system merges these varied approaches to enable the model to recognize pet behaviors with greater accuracy and reliability. The learning process, while based on pseudo labels, integrates data augmentation via UDA and an inactive data identification method, making the model more robust and dependable in its predictions. Figure 4 illustrates this complex learning process, and Figure 5 shows the structure of the proposed teacher and student models in this study.

Augmentation is performed on the preprocessed unlabeled data set. The augmented unlabeled data and the labeled data are combined along the batch dimension for use in the subsequent training process.

The student model’s training utilizes pseudo labels generated by the teacher model. The teacher model makes predictions on the unlabeled data to generate pseudo labels (${\hat{y}}_{u}$). The student model then uses these pseudo labels as if they were actual labels to proceed with its training.

Equation (1) represents the update process of the student model, where $\theta_{S}^{t}$ denotes the current parameters of the student model, and represents the parameters after the update. Cross Entropy calculates the difference between the predictions of the student model and $\theta_{S}^{t+1}$ the pseudo labels and is updated accordingly. This method is akin to supervised learning, where pseudo labels are used as ground truth labels.

(1) $$\theta_{S}^{t+1}=\theta_{S}^{t}-\eta_{S}\;\nabla_{\theta_{S}}CE\left({\hat{y}}_{u},S\left(x_{u};\theta_{S}\right)\right)$$

After the student model is trained, the teacher model is updated through feedback. This teacher loss composition primarily combines MPLs (Meta Pseudo Labels) loss, UDA loss, and distance loss. Equation (2) is used for the meta pseudo labels teacher loss, which calculates the change in the student model. The variable h represents the change in loss and is used to determine the weight.

(2) $$h=\nabla_{\theta_{S}}CE\left(y_{l},S\left(x_{l};\theta_{s}^{\left(t+1\right)}\right)\right)-\nabla_{\theta_{S}}CE\left(y_{l},S\left(x_{l};\theta_{s}^{t}\right)\right)$$

(3) $$L_{MPL}=h\cdot \nabla_{\theta_{T}}CE({\hat{y}}_{u},T\left(x_{u};\theta_{T}\right))$$

Equation (3) represents the MPL loss used to update the teacher model. This loss combines the change in the student model’s loss with the prediction error of the teacher model. Through the MPL loss, the teacher model receives feedback from the student model and updates its parameters accordingly. This feedback enables mutual learning between the student and teacher models, allowing both models to learn through this process.

#### 3.3.1. Unlabeled Data Augmentation for Consistency

To enhance the performance of the model recognizing pet behaviors, Unlabeled Data Augmentation for Consistency Loss was employed in combination. This approach uses contrastive learning between Weak Augmentation and Strong Augmentation to ensure the model maintains consistent predictions across various data transformations. In particular, the teacher model can generate reliable pseudo labels without being overly sensitive to minor changes through this contrastive learning.

Weak Augmentation introduces minor changes to the data, enabling the model to adapt to the variety of changes that might occur in everyday scenarios. Techniques such as Flip and Jittering were utilized in this process. Through these subtle changes, the model can learn more general features.

Strong Augmentation applies more dramatic and complex data transformations, pushing the model to adapt to a broader range of data variations and complex environments. Techniques like Dimension Shuffle and Time Inverse significantly alter the data’s fundamental structure, enabling the model to adapt to strong changes. Dimension Shuffle rearranges the (x, y, z) dimensions of the data, helping the model to extract essential information beyond the basic form and pattern. Time Inverse reverses the temporal order in time-series data, teaching the model to understand temporal changes.

(4) $$L_{cons}=\nabla_{\theta_{T}}CE\left(T\left(x_{uw};\theta_{T}\right),\;T\left(x_{uh};\theta_{T}\right)\right)$$

(5) $$L_{UDA}=\nabla_{\theta_{T}}CE\left(y_{l},T\left(x_{l};\theta_{T}\right)\right)+L_{cons}$$

**Figure 6:**
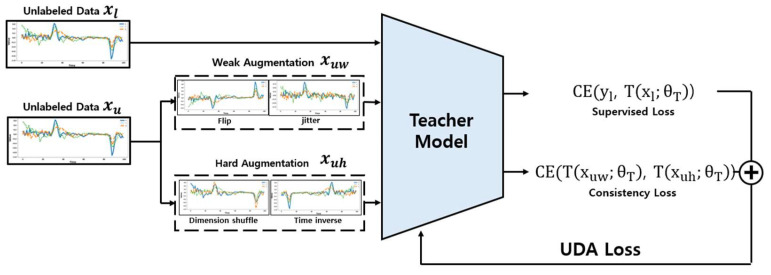
UDA Loss update process using Weak Augmentation and Hard Augmentation.

The overall UDA loss update process is as shown in Figure 6. The augmented data and labeled data are used for predictions by the teacher model, and the UDA loss is calculated based on these predictions. This process is conducted simultaneously with the meta pseudo labels update.

The process of calculating the consistency loss is as described in Equation (4). The consistency loss is obtained by calculating the Cross Entropy between the original and transformed data. In this calculation, unlabeled data $x_{uw}$ with Weak Augmentation and unlabeled data $x_{uh}$ with Strong Augmentation are used. The UDA loss is ultimately calculated as the sum of the Cross Entropy between the teacher model’s predictions for labeled data and the actual label values, and the consistency loss, as expressed in Equation (5). The integrated use of Weak and Strong Augmentation through UDA not only enables the model to effectively learn from unlabeled data but also ensures stable predictions in real-world settings.

#### 3.3.2. Cleaned of Inactive Data

**Figure 7:**
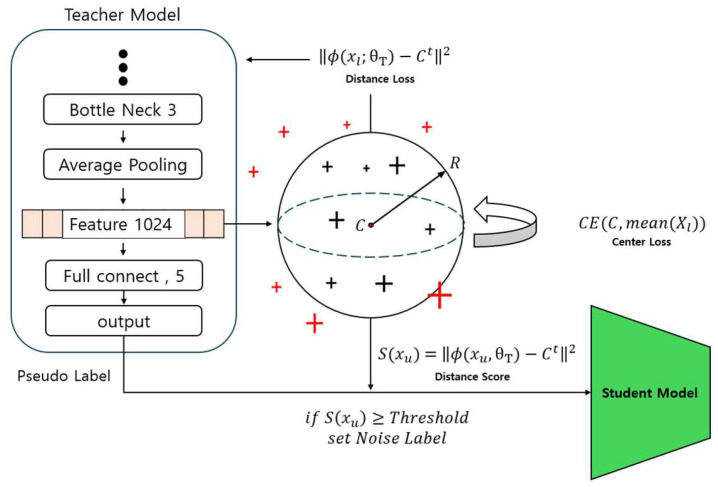
Embedding space used for cleaning inactive data.

Eliminating inactive data, which does not belong to any classification group, is crucial for the model’s rapid convergence and stable performance. However, unlabeled data often contains a significant amount of such inactive data. This paper presents an approach to identify inactive data by employing embedding space compression to remove this inactive data. The added Enhanced Exclusion of Inactive Data process in this paper is depicted in Figure 7.

The teacher model’s neural network structure extracts the feature vector $\phi (x_{l};\theta_{T})$ from the labeled data $x_{l}$. These extracted feature vectors are used to form the embedding space of the labeled data, playing a crucial role in measuring the distances between data points in this space.

(6) $$L_{dis}=\;\;\nabla_{\theta_{T}}{\left\|\phi (x_{l};\theta_{T})-C^{t}\right\|}^{2}$$

(7) $$C^{t+1}=C^{t}-{\eta_{S}\nabla }_{\theta_{T}}CE(C^{t},{mean(X}_{l}))$$

The primary mechanism for compressing the embedding space is defined by the distance loss in Equation (6). This loss function aims to minimize the squared Euclidean distance between each data point’s feature vector and the central value (*C*) of the embedding space. This process makes data points in the embedding space denser, allowing features to be more compact and cohesive. *C* is calculated based on the average of the feature vectors of the labeled data points and serves as a centroid in the embedding space.

The central value is updated according to Equation (7), where the Cross Entropy loss between the current central value and the average value among the data points is calculated, and based on this the central value is updated. This updating process more accurately reflects the central value in the embedding space, serving to construct and optimize the embedding space using the labeled data. Additionally, this embedding space can serve as a criterion for identifying and classifying inactive data included in the unlabeled data.

The Distance Score plays a crucial role in the process of identifying inactive data in unlabeled data. This score represents the distance between the unlabeled data points and the centroid (*C*) of the embedding space and is calculated through Equation (8).

(8) $$S(x_{u})={\left\|\phi \left(x_{u},\theta_{T}\right)-C^{t}\right\|}^{2}\;$$

The Distance Score is defined as the square of the Euclidean distance between the feature vector and the centroid. This distance serves as a criterion for identifying inactive data. If the Distance Score exceeds a threshold, the corresponding unlabeled data are considered inactive data and are relabeled as noise in the teacher model’s output, the pseudo label. This threshold setting and update are based on analyzing the distances among labeled data points, as shown in Equations (9)–(11).

(9) $$\mu_{t+1}=\alpha \cdot S\left(x_{l}\right)+(1-\alpha )\cdot \;\mu_{t}$$

(10) $$\sigma_{t+1}=\alpha \cdot S\left(x_{l}\right)+(1-\alpha )\cdot \;\sigma_{t}$$

(11) $$T_{t+1}=\mu_{t+1}+\beta \cdot \sigma_{t+1}$$

The system utilizes an updating mechanism for the average distance (*μ*) and standard deviation (*σ*) between label pointers. The update of the average is adjusted according to Equation (9), reflecting the Distance Score of new data pointers, while the update of the standard deviation is carried out according to Equation (10). Here, *α*, which plays a role similar to a learning rate, determines the extent of the influence new data have on the average and standard deviation. Subsequently, using the updated average and standard deviation, the threshold value $T_{t+1}$ is calculated as per Equation (11). In this calculation, the adjustment coefficient *β* for the standard deviation is added to the updated average. As the standard deviation tends to decrease over time, β is progressively increased through a scheduler. This approach effectively discriminates inactive data and re-adjusts pseudo labels from noise labels, thereby maintaining data quality and optimizing model performance.

Finally, the Teacher model’s loss function is formed by combining these various components and is updated as described in Equation (12).

(12) $$\theta_{T}^{t+1}=\theta_{S}^{t}-\eta_{S}\;\cdot (L_{MPL}+L_{UDA}+L_{dis})\;$$

In meta pseudo labels, the model integrates feedback from the student model, contrastive learning through UDA, supervised learning, and Distance Loss for removing inactive data. Each of these loss components guides the model’s learning in different directions, and the combined loss function enables the model to learn from unlabeled data more intricately.

The final learning structure of this study is conducted through the following processes. Initially, the teacher model generates pseudo labels, which are then identified and revised using the embedding space to distinguish and correct noise labels. The revised pseudo labels are updated through the student model using Cross Entropy. During this process, the student model receives various losses, including MPL loss, UDA loss based on contrastive learning, and distance loss, which measures the distance between vectors and the center in the embedding space. These losses are combined and utilized as a comprehensive loss for training the student model. Through this method, the model achieves more refined and efficient learning, ultimately reaching higher accuracy and reliability. The pet behavior recognition system model was updated in this manner and its performance was measured through various experiments and metrics.

## 4. Experiment

### 4.1. Experimental Setup

This study was implemented using Python and the PyTorch framework. All experiments were conducted in a consistent environment, and the detailed specifications used for the experiments are listed in Table 1. This consistent experimental setup enhances the reliability of the research results and minimizes variables when comparing with other studies.

### 4.2. Data Configuration

This study conducted data collection on a total of 12 pets of various types and sizes. Detailed information on each animal, such as breed, age, and weight, is systematically presented in Table 2. The data were collected at a sampling rate of 100 Hz, capturing the behavior effectively. These data were gathered using specially designed collars equipped with sensors, which were comfortably worn around the pets’ necks. Additionally, throughout the data collection process, special care was taken to ensure that the animals remained with their owners to prevent any distress. This approach not only facilitated a smooth data collection process but also ensured the well-being and comfort of the participating pets.

Some of the collected data were utilized as labeled data, while the rest were used as unlabeled data. The unlabeled data were structured using a slicing window based on a 50 Hz sampling rate. The final count of labeled data by label and the quantity of unlabeled data used in the experiments are presented in Table 3.

### 4.3. 4D Reshaping Supervised

A simple experiment was conducted to compare the performance between 1-dimensional and 4-dimensional structures using conventional supervised learning. ResNet, a type of residual network introduced to solve the vanishing gradient problem that can occur in deep neural networks, has the capability to effectively learn complex features [27]. The performance comparison results using ResNet are as shown in Table 4, demonstrating that the 1-dimensional structure outperforms the 4-dimensional structure. This indicates that the preprocessing approach for the 4-dimensional structure is more suitable for processing sensor data in this study.

### 4.4. Pseudo Label Experiment Result

An experiment was conducted to investigate the effectiveness of the meta pseudo labels method for pet behavior recognition using sensor data. In the experiment, learning was carried out using only labeled data. Subsequently, a certain percentage of the labeled data set was randomly selected and assumed to be unlabeled data for the experiment. The batch size was set to 50, and the learning was conducted for 50,000 steps. The experimental results are as shown in Table 5. Since labeled data do not contain inactive data, the experiment was conducted by combining meta pseudo labels with UDA loss.

According to the results of the comparison experiments, the supervised method achieved an accuracy (ACC) of approximately 82.93%. In contrast, the noise student method showed better performance than supervised learning in over 60% of the data. However, when the labeled data were below 40%, there was a decline in performance. Additionally, the noise student method recorded lower performance in the F1_Score metric in all cases compared to supervised learning, which could indicate a drop in the balance between precision and recall for this method compared to supervised learning.

On the other hand, the meta pseudo labels method showed similar or better performance than supervised learning even when using only 20% of labeled data. Notably, when combined with UDA loss, the performance of this method improved, achieving an accuracy of about 83.36%. When the labeled data were more than 40%, the meta pseudo labels method exhibited superior results in all cases compared to supervised learning.

**Figure 8:**
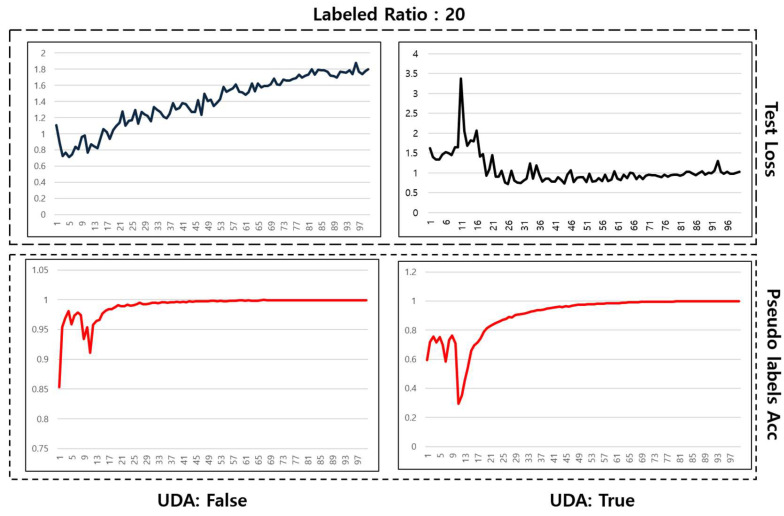
Learning graph with label data ratio of 20%.

Figure 8 compares the meta pseudo labels method with and without the application of UDA when labeled data constitute 20% of the total. The top-left graph, which represents test loss without UDA, shows an increasing trend during the learning process, indicating signs of overfitting and a tendency for the loss to diverge. In contrast, the top-right graph illustrates that test loss becomes much more stable and converges when UDA is applied, with a significant reduction in divergence. This suggests that UDA effectively enhances the model’s generalization capability, preventing overfitting.

The bottom-left and bottom-right graphs show the trends in the accuracy of pseudo labels without and with the application of UDA, respectively. Both graphs exhibit variability in the early stages of learning, but a trend of increasing accuracy over time can be observed.

Overall, these experimental results demonstrate that the meta pseudo labels method can effectively utilize a small number of labeled data and offer a potent alternative to supervised learning for analyzing pet behavior. However, it’s important to note that this study did not use actual unlabeled data but instead separated and used labeled data, which may limit the interpretation of the results in the context of real unlabeled data.

### 4.5. Cleaned Meta Pseudo Labels Experiment Results

Table 6 presents the results of an experiment conducted using actual unlabeled data, employing the cleaned meta pseudo labels approach, which integrates MPL loss, UDA loss, and distance loss.

**Figure 9:**
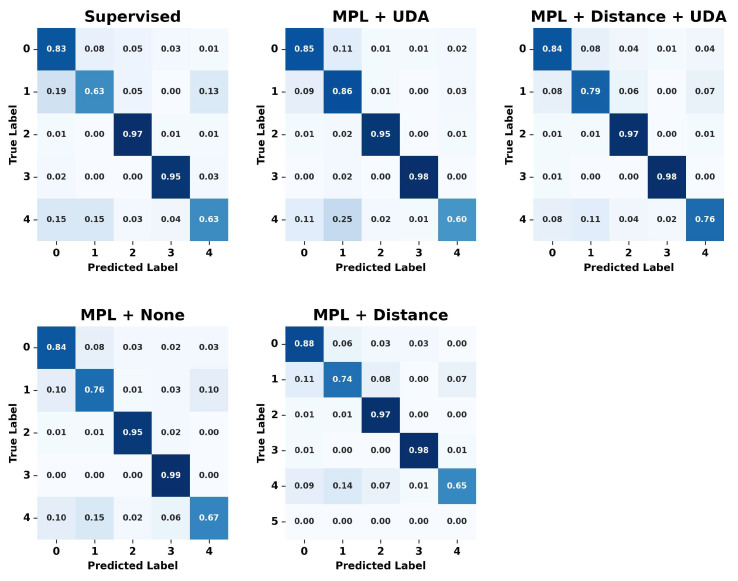
Pseudo labels learning results confusion matrix using real unlabeled data.

The experiment demonstrated that the cleaned meta pseudo labels model achieved the highest accuracy of 88.31%, and the F1_Score was also high at 87.12%, proving the effectiveness of the learning process. This indicates that noise removal helps in refining the data and can enhance the model’s performance. Compared to traditional supervised learning, the cleaned meta pseudo labels method, with its combined loss function, showed lower test loss and higher accuracy. Figure 9 displays the confusion matrices of the predictions made by each model, providing insight into their performance across different classes.

In the confusion matrix, each row represents the actual label, while each column represents the label predicted by the model. Thus, the values along the diagonal represent the proportion of instances that were correctly classified for each label, and the off-diagonal values indicate the proportion of misclassifications. The implementation of UDA and the removal of inactive data resulted in performance improvements across all labels, indicating that the combination of these learning techniques outperforms traditional supervised learning methods and creates a model that is robust to various transformations. The cleaned meta pseudo labels method proposed in this study demonstrated effective performance in classifying various behavioral patterns by integrating UDA loss, distance loss, and MPL loss. Notably, it achieved superior results even in categories such as ‘eating’ behavior, which other learning methods find challenging to classify. This success can be attributed to the proposed learning method’s ability to maintain high accuracy through the process of removing noise from unlabeled data.

**Figure 10:**
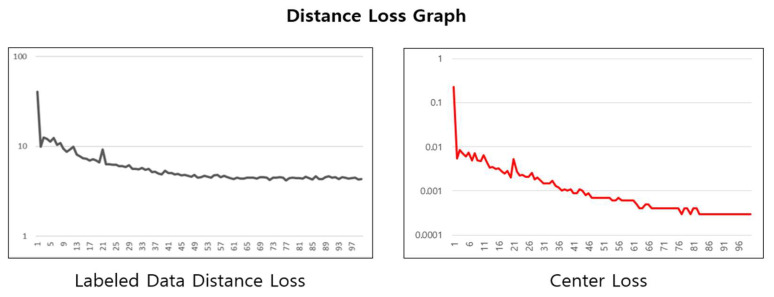
Cleaned meta pseudo labels learning graph for distance loss.

Figure 10 illustrates the changes in two types of loss functions during the cleaned meta pseudo labels learning process. The left graph shows the changes in the distance loss for labeled data, and the right graph displays the changes in the center loss.

The labeled data distance loss graph shows a trend of gradual decrease and stabilization over time, indicating that the distance loss is converging as the learning progresses, and the learning based on the loss function is being effectively applied. This demonstrates that the embedding space formation and compression for labeled data are being learned, and by effectively removing noise data, the performance of the learning has been enhanced.

The experimental results indicate that in the pet behavior recognition system, the method combining MPL and UDA outperformed traditional supervised learning approaches. Furthermore, the cleaned meta pseudo labels learning method proposed in this paper has been confirmed to provide good prediction performance in time-series data containing inactive data.

## 5. Conclusions

In this study, pet behaviors are recognized using a total of 12 axes of data, including acceleration, magnetometer, gyroscope, and rotation values. Five behaviors were predicted: standing, walking, sitting, lying down, and eating, with a 2-s action time frame for predictions.

The data set was collected using a wearable device positioned under the pet’s neck, with data collection and storage facilitated through Bluetooth and Wi-Fi communications. The data collection frequency was set at 50Hz, and preprocessing steps such as missing data handling, conversion to a 4D shape, and separation into labeled and unlabeled data were conducted to utilize the data set for cleaned meta pseudo labels learning. However, the unlabeled data often contained a significant amount of inactive data, which, due to the nature of classification models, can degrade the model’s performance if it falls outside the defined categories.

This study aimed to separate inactive data by applying feature value embedding space matching and compression techniques used in the teacher model. The inactive data removed were relabeled as inactive by the teacher model, which then guided the training of the student model. Additionally, by incorporating UDA, a comprehensive learning approach combining meta pseudo labels and inactive data removal was formulated.

The experimental results demonstrated that this combined learning approach exhibited superior performance, achieving a score of 88.31% (ACC). Notably, it showed promising prediction capabilities in the challenging category of ‘eating’.

Future research aims to predict behaviors using a broader range of categories and more extensive data sets and to improve performance by leveraging metadata about the pets in addition to sensor data. Therefore, the intention is to expand this study to involve a more diverse population of pets.

## Figures and Tables

**Table 1:** Experimental specifications.

Metric	Description
CPU	AMD Ryzen 5800X
GPU	NVIDIA RTX 3090(2way)
RAM	64 GB
CUDA	11.8
cuDNN	8.1
Torch	2.2.1
Python	3.8.6

**Table 2:** Basic experimental subject information.

No	Breed	Age (Month)	Weight (kg)
1	Yorkshire terrier	48	7.8
2	Toy poodle	76	4.7
3	Toy poodle	150	5
4	Toy poodle	130	1.5
5	Mini poodle	68	2.2
6	Mini bichon	94	3.1
7	Mix dog	36	4.6
8	Border Collie	24	14.5
9	Mix dog	24	6.3
10	Mix dog	16	15.7
11	Mix dog	24	20.3
12	Mix dog	12	13.2

**Table 3:** Configuration data set.

No. (Label)	Behavior	Number of Data	Proportion of Data	Number of Training Data	Number of Test Data
0	Stand	2007	21.11%	6536	1635
1	Walk	1414	14.90%		
2	Sit	2309	24.33%		
3	Lying	2101	22.14%		
4	Eat	1656	17.52%		
Labeled Total		9487	100%	80%	20%
Unlabeled		11,008	100%	100%	0%

**Table 4:** Comparison of 1D and 4D learning outcomes compared to supervised learning.

	Dimension	Accuracy (%)	Recall (%)	F1_Score (%)
ResNet18	1D	80.48	78.62	79.54
ResNet18	4D	81.28	75.57	78.32
ResNet30	1D	82.00	77.26	79.57
ResNet30	4D	82.43	78.33	80.33
ResNet50	1D	82.46	76.47	79.56
ResNet50	4D	82.93	78.15	80.47

**Table 5:** Meta pseudo labels result using labeled data.

Type	Labeled Data Ratio	UDA	Test Loss	ACC (%)	F1_Score (%)
Noise Student [13]	20%	X	1.42	68.53	26.35
	40%	X	0.86	77.54	56.56
	60%	X	0.76	83.86	67.84
	80%	X	0.65	86.36	76.69
Meta Pseudo Labels [12]	20%	О	0.71	83.36	81.04
	20%	X	1.15	82.56	80.28
	40%	О	0.56	86.11	84.34
	40%	X	0.60	85.68	83.75
	60%	О	0.44	87.18	86.186
	60%	X	0.50	86.91	85.34
	80%	О	0.40	88.19	86.56
	80%	X	0.61	87.21	85.54
Supervised	100%	None	0.58	82.93	80.47

**Table 6:** Remove noise unlabeled data experiment results.

Type	UDA	Inactive Data	Test Loss	ACC (%)	F1_Score (%)
Meta Pseudo Labels	О	О	0.53	88.31	87.12
	X	О	0.45	86.85	84.56
	О	X	0.46	86.91	85.04
	X	X	0.44	86.23	84.32
Supervised	X	X	0.58	82.93	80.47

## Data Availability

The data presented in this study are available from the corresponding author upon request. The data are not publicly available due to privacy and ethical concerns.
